# Functional Characterization of Eight Zinc Finger Motif-Containing Proteins in *Toxoplasma gondii* Type I RH Strain Using the CRISPR-Cas9 System

**DOI:** 10.3390/pathogens12101232

**Published:** 2023-10-11

**Authors:** Jin Gao, Xiao-Jing Wu, Xiao-Nan Zheng, Ting-Ting Li, Yong-Jie Kou, Xin-Cheng Wang, Meng Wang, Xing-Quan Zhu

**Affiliations:** 1 Laboratory of Parasitic Diseases, College of Veterinary Medicine, Shanxi Agricultural University, Taigu, Jinzhong 030801, China; jingao2022@163.com (J.G.); wuxiaojing2017@163.com (X.-J.W.); zhengxiaonan8889@126.com (X.-N.Z.); kouyongjie1997@163.com (Y.-J.K.); 2 State Key Laboratory for Animal Disease Control and Prevention, Key Laboratory of Veterinary Parasitology of Gansu Province, Lanzhou Veterinary Research Institute, Chinese Academy of Agricultural Sciences, Lanzhou 730046, China; litt866@163.com (T.-T.L.); wangxincheng55555@163.com (X.-C.W.); 3 Key Laboratory of Veterinary Public Health of Higher Education of Yunnan Province, College of Veterinary Medicine, Yunnan Agricultural University, Kunming 650201, China

**Keywords:** *Toxoplasma gondii*, toxoplasmosis, zinc finger protein, CRISPR-Cas9, replication

## Abstract

The Zinc finger protein (ZFP) family is widely distributed in eukaryotes and interacts with DNA, RNA, and various proteins to participate in many molecular processes. In the present study, the biological functions of eight ZFP genes in the lytic cycle and the pathogenicity of *Toxoplasma gondii* were examined using the CRISPR-Cas9 system. Immunofluorescence showed that four ZFPs (RH248270-HA, RH255310-HA, RH309200-HA, and RH236640-HA) were localized in the cytoplasm, and one ZFP (RH273150-HA) was located in the nucleus, while the expression level of RH285190-HA, RH260870-HA, and RH248450-HA was undetectable. No significant differences were detected between seven RHΔ*zfp* strains (RHΔ*285190*, RHΔ*248270*, RHΔ*260870*, RHΔ*255310*, RHΔ*309200*, RHΔ*248450*, and RHΔ*236640*) and the wild-type (WT) strain in the *T. gondii* lytic cycle, including plaque formation, invasion, intracellular replication, and egress, as well as in vitro virulence (*p* > 0.05). However, the RHΔ*273150* strain exhibited significantly lower replication efficiency compared to the other seven RHΔ*zfp* strains and the WT strain, while in vivo virulence in mice was not significantly affected. Comparative expression analysis of the eight *zfp* genes indicates that certain genes may have essential functions in the sexual reproductive stage of *T. gondii*. Taken together, these findings expand our current understanding of the roles of ZFPs in *T. gondii*.

## 1. Introduction

*Toxoplasma gondii* is an obligate intracellular apicomplexan of significant medical and veterinary importance, infecting approximately 30% of the world’s population [1]. *T. gondii* infections often appear asymptomatic in immunocompetent individuals but can pose life-threatening risk in immunocompromised individuals (such as people with AIDS), and most severe cases are caused by recrudescent infections [2,3]. In addition, *T. gondii* infection during pregnancy may cause abortion or severe congenital toxoplasmosis in the newborn [1,4]. Unfortunately, the current treatments for toxoplasmosis have adverse reactions and cannot cure chronic infections [5].

Zinc finger proteins (ZFPs) represent a large group of proteins that contain one or more zinc finger domains [6]. The zinc finger domain is characterized by the coordination of zinc ions by specific cysteine and histidine residues [7], and the domain can bind to DNA, RNA, or other proteins, allowing zinc finger proteins to participate in a wide range of cellular processes, including gene regulation, DNA repair, RNA metabolism, and protein–protein interactions [8,9]. Recently, the role of ZFPs in the pathogenicity of *T. gondii* has attracted increasing interest. For example, *T. gondii* ZFP1 contains three CCHC zinc finger motifs and plays an important regulatory role in tachyzoite-to-bradyzoite differentiation [10]. A *T. gondii* ZF domain-containing protein (TgZFP2) plays a significant role in the mitosis process of *T. gondii* and is essential for its growth in vitro [11]. Inducible knockout of the TgZNF2 protein, which possesses a conserved C2H2 zinc finger motif, resulted in G1 phase inhibition in the mutant strain’s cell cycle. Further investigations have demonstrated that this protein is essential for the nuclear export of polyA + mRNA in *T. gondii* [12]. Bradyzoite Formation Deficient 1 (BFD1) is a major regulatory factor in the chronic differentiation of *T. gondii* [13]. A recent study has revealed that CCCH-type zinc finger protein (BFD2) is the second indispensable factor in the chronic stages of infection, which is in a positive feedback loop with BFD1, and its absence decreases the protein level of BFD1 [14]. This discovery represents a significant advancement in the understanding of ZFPs in *T. gondii*. Nevertheless, the functions of the majority of ZFPs remain unknown.

To better understand the biological function of ZFPs in *T. gondii*, we studied the function of the eight selected *zfp* genes (TGME49_285190, TGME49_248270, TGME49_260870, TGME49_255310, TGME49_309200, TGME49_248450, TGME49_236640, and TGME49_273150) utilizing the CRISPR-Cas9 system. Following the successful C-terminal endogenous tagging of eight ZFPs, the subcellular localization of eight ZFPs was determined in the tachyzoite stage. The pathogenicity of these eight RHΔ*zfp* strains was assessed via plaque assay, invasion, intracellular replication, calcium ionophore-induced egress assay, and acute virulence assay in mice. In addition, transcriptome data for the eight *zfp* genes were analyzed to further understand their dynamic expression in different stages of the life cycle.

## 2. Materials and Methods

### 2.1. Bioinformatics Analysis of zfp Genes

The obtained genomic data, including the number of exons, CRISPR-Cas9 phenotype values, transmembrane helices, molecular weights, signal peptides, and the transcriptomic data including cell cycle expression profiles (RH), transcriptional profiles of *zfp* genes in different genotypes, and developmental expression profiles in oocysts, tachyzoites, and bradyzoites for the *zfp* genes were retrieved from ToxoDB (http://toxodb.org, accessed on 3 October 2023) [15].

### 2.2. Parasite Strains

*T. gondii* tachyzoites (Type I RH∆*ku80* and RH∆*zfp* knockout strains) were maintained in confluent monolayers of human foreskin fibroblasts (HFFs, ATCC, Manassas, VA, USA) cultured in Dulbecco’s modified Eagle medium (DMEM) supplemented with 2% fetal bovine serum (FBS), 10 mM HEPES (pH 7.2), 100 μg/mL streptomycin, and 100 U/mL penicillin at 37 °C with 5% CO_2_. The tachyzoites were purified using a 3 µm polycarbonate membrane filter as previously described [16].

### 2.3. Construction of Epitope Tagging Strains

To investigate the expression and localization of ZFPs in the tachyzoite stage of the RH strain, we performed C-terminal endogenous tagging for eight ZFPs. CRISPR plasmid targeting the 3′-untranslated region (3′-UTR) of the *zfp* gene was obtained as previously described [17]. A short homology fragment containing 6 × HA tag products and DHFR fragments was amplified from the pLIC-6×HA-DHFR plasmid using specific primers located near the SgRNA at the 3′ end of the corresponding *zfp* gene. The purified fragment was co-transfected with successfully sequenced plasmid into RH∆*ku80* strain. After selection with pyrimethamine, the positive single clones were confirmed using Polymerase Chain Reaction (PCR), followed by verification via the application of immunofluorescence analysis (IFA) and Western blotting. Table S1 contains the primers employed in the construction of C-terminal epitope tagging strains.

### 2.4. CRISPR-Cas9 Mediated Knockout of zfp Genes

The candidate *zfp* genes were deleted by CRISPR-Cas9-mediated homologous recombination technology as described previously [18]. The UPRT targeting guide RNA (gRNA) in the pSAG1:CAS9-U6:sgUPRT plasmid was replaced with corresponding guide RNAs to construct the candidate *zfp* gene-specific CRISPR plasmids. To obtain 5′-UTR-DHFR-3′-UTR homologous templates of *zfp* genes, the ~1 kb homology arms of 5′-UTR and 3′-UTR were amplified by using *T. gondii* genomic DNA as the template, and the DHFR drug-resistant fragment was amplified from the pUPRT-DHFR-D plasmid. The pUC19 fragment was amplified from the pUC19 plasmid and used as the vector for cloning the above fragments using the CloneExpress II one-step cloning kit (Vazyme, Nanjing, China). The positive plasmid served as a template for amplifying the 5′-UTR -DHFR-3′-UTR homologous fragment. The primers used for constructing the knockout plasmids and homologous templates can be found in Table S2.

For transfection, about 35 μg *zfp* gene-CRISPR-Cas9 specific knockout plasmid and ~20 μg purified 5′-UTR -DHFR-3′-UTR homologous fragment was co-transfected into purified tachyzoites of RHΔ*ku80*. Following three rounds of drug selection with 3 µM pyrimethamine, the single clones were isolated via limiting dilution in 96-well plates. Subsequently, the *zfp* knockout strains were identified with genome DNA level using diagnostic PCRs.

### 2.5. Immunofluorescence Assay and Western Blotting Analysis

IFA was conducted to determine the localization of ZFPs in the *T. gondii* RH strain as previously described [19]. Briefly, freshly egressed tachyzoites were grown in confluent monolayers of HFFs for 24 h. Afterward, the cells were fixed with 4% paraformaldehyde (PFA) and permeabilized with 0.1% Triton X-100 for 20 min. The primary antibodies (rabbit anti-IMC1 and mouse anti-HA (Invitrogen, Thermo Fisher Scientific, Waltham, MA, USA) were applied at 1:500 dilutions and incubated at 37 °C for 2 h. Following five washes, the secondary antibodies [Alexa Fluor 488 goat anti-rabbit IgG (H + L) (1:1000) and Alexa Fluor 594 goat anti-mouse IgG (1:1000)] were added to HFF monolayers at 37 °C for 1 h. The nuclei were stained with 4′, 6-diamidino-2-phenylindole (DAPI), and the samples were visualized using the Leica confocal microscope system (TCS SP8, Leica, Munich, Germany).

For the Western blotting analysis, the freshly egressed tachyzoites were washed twice with sterile, cold PBS and lysed on ice with RIPA lytic buffer containing protease inhibitor and EDTA for 30 min, followed by centrifugation to obtain the supernatants. The supernatants were mixed with 4× loading buffer and boiled for 10 min. The resulting protein samples were separated on 10% polyacrylamide gels and transferred onto a polyvinylidene fluoride (PVDF) membrane. The antibodies used in Western blot include rabbit anti-aldolase (1:500), rabbit anti-HA (1:500), and goat anti-rabbit HRP (1:500). Protein signals were detected using an ECL chemiluminescence kit (Thermo Fisher Scientific, Waltham, MA, USA) as described previously [20].

### 2.6. Parasite Plaque Assay

For the plaque assay, HFF monolayers in a 12-well cell plate (Thermo Fisher Scientific) were infected with freshly egressed RHΔ*zfp* strains and WT strain (approximately 500 tachyzoites per well). The plates were incubated at 37 °C, 5% CO_2_ for 7–9 days without any interference. Then, the culture medium was removed, and the infected monolayers were fixed with 4% PFA. For plaque visualization, the sample was stained with 0.2% crystal violet for a duration of 20 min. The size and number of plaques per well were analyzed using the ImageJ 1.46 software. Three biology replicates were performed for each sample.

### 2.7. Invasion Assay

To examine whether *zfp* genes contribute to the invasion efficiency of *T. gondii*, approximately 2 × 10^6^ freshly egressed tachyzoites (RHΔ*zfp* strains and WT strain) were permitted to invade the HFF monolayers in a 12-well cell plate (Thermo Fisher Scientific) at 37 °C for 30 min. Then, the culture medium was removed, and 4% PFA was added for fixation. Before permeabilization, extracellular parasites were stained with mouse anti-SAG1 antibody (1:500) at 37 °C for 2 h and then with Alexa Fluor 594 goat anti-mouse antibody (1:500) at 37 °C for 1 h. After three washes with PBS, infected HFF monolayers were permeabilized with 0.1% Triton X-100 for 30 min. For all parasites, the infected cells were stained with rabbit anti-GAP45 antibody (1:500) at 37 °C for 2 h, followed by staining with Alexa Fluor 488 goat anti-rabbit antibody (1:500) at 37 °C for 1 h, as described previously [21]. Using a fluorescence microscope, the invasion of tachyzoites was examined, with uninvaded tachyzoites stained in red and the total tachyzoites stained in green.

### 2.8. Intracellular Replication and Calcium Ionophore-Induced Egress Assay

To assess potential differences between the RHΔ*zfp* strains and WT strain in the processes of intracellular replication and egress, the tachyzoite replication and calcium ionophore-induced egress assay were conducted as previously described [21]. Freshly egressed tachyzoites (1 × 10^5^) from RHΔ*zfp* strains and WT strain were inoculated into HFF monolayers in 12-well plates (Thermo Fisher Scientific). After 1 h, the uninvaded tachyzoites were washed away with a pre-warmed DMEM medium. For the intracellular replication assay, infected HFF monolayers were cultured at 37 °C for another 23 h and fixed with 4% PFA for 30 min. After permeabilization, the samples were stained with mouse anti-SAG1 antibody, followed by Alexa Fluor 488 goat anti-mouse IgG. The number of tachyzoites in at least 200 parasitophorous vacuoles (PVs) was assessed for each sample using a fluorescence microscope. All samples were performed for three independent experiments.

For the egress assay, infected HFF monolayers were incubated for another 36 h. Following the removal of the culture medium, DMEM containing 3 µM of the calcium ionophore A23187 (Sigma, Burlington, MA, USA) was introduced. After a 2 min interval, the dish was promptly fixed. The percentage of PVs with or without tachyzoite egress was stained with SAG1 and then counted. Each assay was performed in triplicate.

### 2.9. Assessment of the Virulence in Mice

Female Kunming mice aged six to eight weeks were purchased from the Center of Laboratory Animals of Lanzhou Veterinary Research Institute, Chinese Academy of Agricultural Sciences. The mice were housed in a controlled room maintained under pathogen-free conditions, with a consistent 12 h dark/light cycle, 50–60% humidity, and a temperature of 22 °C. The mice were accessible ad libitum to sterilized water and food. The mice underwent a one-week acclimation period before the experiment.

Mice were infected by intraperitoneal (i.p.) injection with 50 or 100 freshly egressed tachyzoites of RHΔ*zfp* strains and WT strain. Additionally, an equivalent number of tachyzoites used for injection was inoculated for a parallel plaque assay to assess their viability. The infected mice were monitored at least twice a day, and their survival rates were recorded throughout the experiment. Mice showing irreversible signs of death were euthanized to prevent further suffering as described previously [19].

### 2.10. Statistical Analysis

Statistical comparisons were conducted using GraphPad Prism version 8.4.0. All data were analyzed by two-tailed unpaired Student’s *t*-test and one-way analysis of variance (ANOVA). Values are means ± SD for three independent experiments. The standard errors of the means were represented by error bars. *p*-value < 0.05 indicates that the data between infection and control groups exhibit statistical significance.

## 3. Results

### 3.1. Bioinformatics Characteristics of Eight zfp Genes in T. gondii

**Figure 1:**
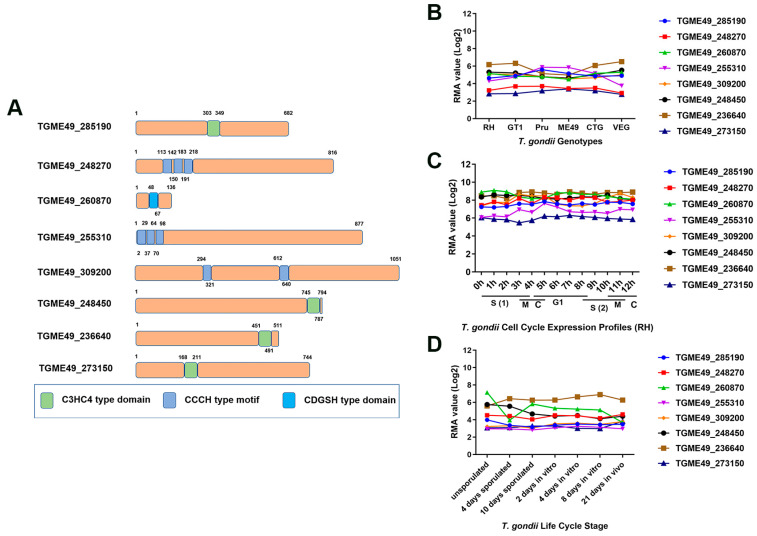
Bioinformatics analysis of selected zinc finger motif-containing Proteins (ZFPs) in *Toxoplasma gondii*. (**A**) The models of ZFPs predicated by SMART (Simple Modular Architecture Research Tool) (http://smart.embl.de, accessed on 3 October 2023) and NCBI’s Conserved Domain Database (CDD) (https://www.ncbi.nlm.nih.gov/Structure/cdd/cdd.shtml, accessed on 3 October 2023) showed that eight ZFPs contain the C3HC4-type domain, CCCH-type motif, or CDGSH-type domain. (**B**–**D**) The transcriptomic data for *zfp* genes were obtained from ToxoDB (http://ToxoDB.org, accessed on 3 October 2023), including transcriptional profiles of *zfp* genes in different genotypes (**B**), different cell cycle expression profiles (RH) (**C**), and developmental expression profiles of parasite life cycle stages (**D**).

The bioinformatics characteristics of eight *zfp* genes are summarized in Table 1. In this study, three genes (TGME49_248270, TGME49_255310, and TGME49_309200) encode C3HC4-type zinc finger domain-containing proteins, while TGME49_285190, TGME49_248450, TGME49_236640, and TGME49_273150 encode CCCH-type zinc finger motif-containing proteins. Additionally, the gene TGME49_260870 encodes a protein with a CDGSH-type zinc finger domain (Figure 1A). These *zfp* genes encode proteins of varying sizes (21.367~101.661 kDa) with different exons. Among them, TGME49_24845 had the highest number of exons (eight exons), while TGME49_309200 had the lowest number of exons (two exons). Except for TGME49_248450 and TGME49_236640, the other *zfp* genes did not have transmembrane domains. Additionally, none of the eight *zfp* genes had a signal peptide. To investigate the expression patterns of these genes, transcriptome data were obtained from ToxoDB. Among the eight *zfp* genes, only TGME49_255310 exhibited differential transcription levels in different genotypes (type I, type II, and type III) of *T. gondii* (Figure 1B). Analyzing the expression profile of the different genes in the cell cycle stages, we observed that none of the eight *zfp* genes followed a cell cycle expression pattern (Figure 1C). TGME49_260870 exhibited differential expression during different developmental stages of *T. gondii*, while the other seven *zfp* genes showed a tendency to be constitutively expressed genes (Figure 1D).

### 3.2. Subcellular Localization of ZFPs

**Figure 2:**
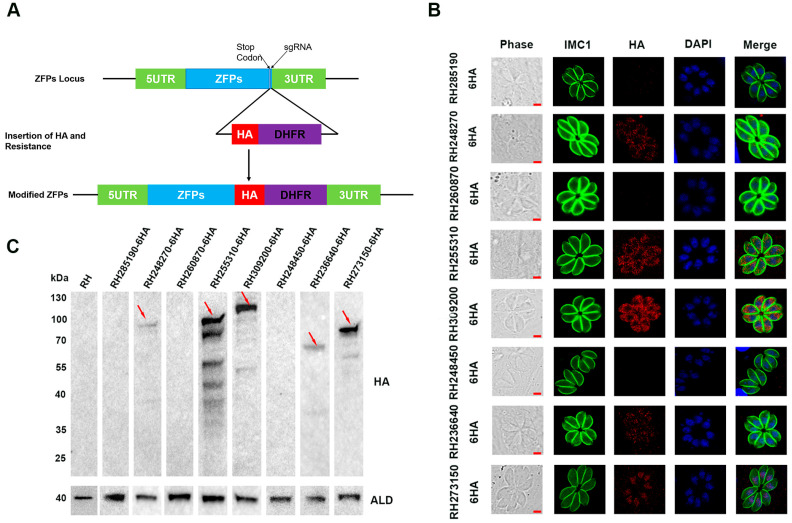
Construction of epitope tagging strains and subcellular localization of eight ZFPs in *Toxoplasma gondii* tachyzoites. (**A**) Schematic illustration showing the C-terminal endogenous tagging for eight ZFPs by using the CRISPR-Cas9 system. (**B**) HFF cells were infected with C-terminally HA epitope tagging strains for 24 h and stained with anti-IMC1 (green) antibody and anti-HA (red) antibody. Nuclei were labeled with DAPI. Scale bars, 2 μm. (**C**) Western blotting was performed to confirm the expression of the eight 6 × HA-tagged proteins in RH strains. Anti-aldolase (ALD) served as a loading control.

To determine the subcellular localization of the eight ZFPs in *T. gondii* type I RH strain, a 6 × HA tag was inserted at the C-terminal using the CRISPR-Cas9 system (Figure 2A). DNA sequencing confirmed the successful insertion of the 6 × HA tag into all eight ZFPs. IFA was performed to examine the subcellular localization of the tagged proteins. The results showed that RH248270-HA, RH255310-HA, RH309200-HA, and RH236640-HA were localized in the cytoplasm, with RH236640-HA showing relatively weak fluorescence (Figure 2B). The expression RH273150-HA was detected in the nucleus, as indicated by co-staining with the nuclear marker DAPI (Figure 2B). Nevertheless, no fluorescence signal was detected for RH285190-HA, RH260870-HA, and RH248450-HA.

To further confirm the expression of the tagged proteins, Western blotting was performed using an anti-HA antibody. The results demonstrated that RH248270-HA, RH255310-HA, RH309200-HA, RH236640-HA, and RH273150-HA exhibited bands of the expected size; among them, RH255310-HA and RH309200-HA displayed multiple bands in addition to the target band. In contrast, no band was detected in the RH285190-HA, RH260870-HA, and RH248450-HA strains, which is consistent with the fluorescence results, indicating that these proteins may not be expressed or may be present at extremely low levels in the tachyzoites stage (Figure 2C).

### 3.3. Successful Deletion and Identification of ZFPs in the T. gondii Type I RH Strain

**Figure 3:**
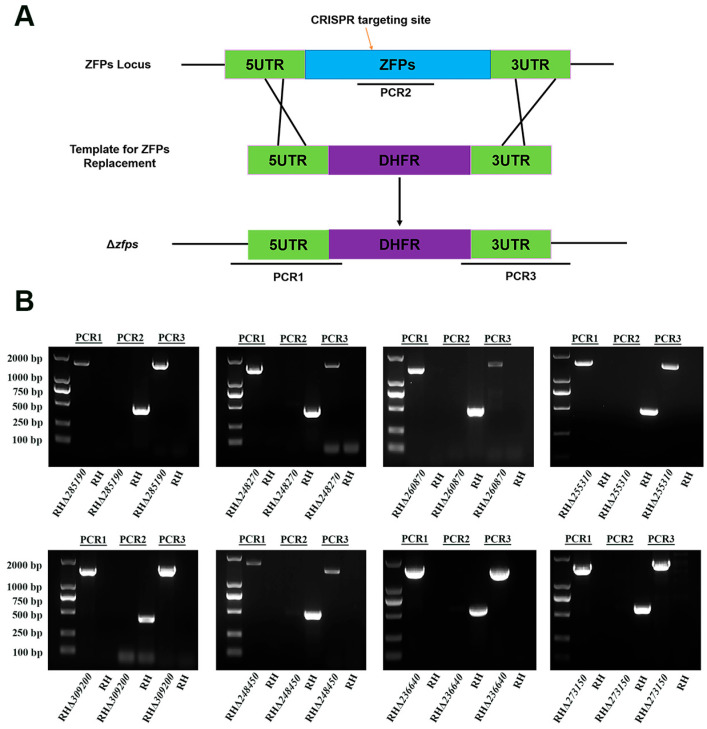
Construction of eight RHΔ*zfp* strains by CRISPR-Cas9. (**A**) Schematic illustration showing strategy used to construct the eight *zfp* genes knockout strains of *T. gondii* RH strain by replacing the coding region of each *zfp* gene. (**B**) Polymerase chain reactions (PCRs) were used to identify successful deletion of *zfp* genes in *T. gondii* RH strain. PCR1 and PCR3 were used to examine the insertion of the 5′ and 3′ homologous fragments, whereas PCR2 was used to detect the successful deletion of *zfp* genes.

To investigate the biological functions of *zfp* genes in the *T. gondii* type I RH strain, the coding region of each *zfp* gene was replaced by a corresponding homologous fragment (5′ UTR-DHFR-3′ UTR) using the CRISPR-Cas9 system (Figure 3A). The single clone was obtained by limiting dilution and pyrimethamine selection. PCRs were performed to confirm the successful knockout of *zfp* genes. The successful insertion of the homologous fragments into the target gene locus was validated using PCR1 and PCR3, in which ~1000–1500 bp fragments were amplified in each *zfp* gene strain (Figure 3B). The expected target fragments (~500 bp) in the coding sequence were detected using PCR2 in the WT strain, whereas no fragments were amplified in the knockout strains (Figure 3B). These results demonstrated the successful deletion of *zfp* genes in the *T. gondii* type I RH strain using the CRISPR-Cas9 system.

### 3.4. TGME49_273150 Is Required for the Growth of T. gondii Type I RH Strain in Vitro

**Figure 4:**
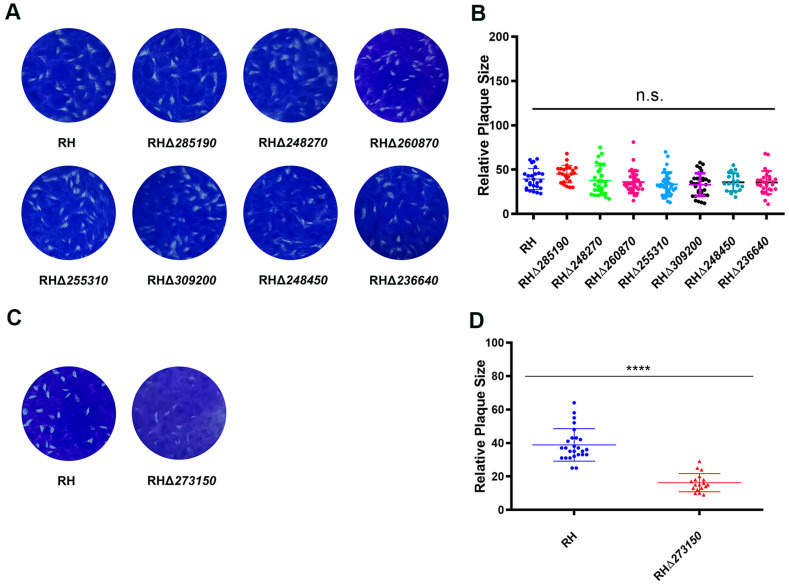
Comparing the lytic cycle of RHΔ*zfp* strains and WT strain in vitro. (**A**) Plaque assay demonstrating no significant differences in the growth between the seven RHΔ*zfp* strains and the WT strain. (**B**) Relative size of plaques produced by the seven RHΔ*zfp* strains and the WT strain. (**C**) Representative images of the plaque assays showing the growth defects when TGME49_273150 was depleted. (**D**) The number and size of plaques formed by RHΔ*273150* strain showed significant reduction compared with WT strain. ****, *p* < 0.0001, n.s., not significant.

To further assess the impact of the deletion of the eight *zfp* genes on the overall growth of the RH strain, a plaque assay on HFF monolayers was conducted. The results showed that there were no significant differences in the number and size of plaques between the seven RHΔ*zfp* strains (RHΔ*285190*, RHΔ*248270*, RHΔ*260870*, RHΔ*255310*, RHΔ*309200*, RHΔ*248450,* and RHΔ*236640*) and the WT strain (*p* > 0.05) (Figure 4A,B). However, the RHΔ*273150* strain exhibited a significant reduction in plaque size (*p* < 0.05) (Figure 4C,D), suggesting that TGME49_273150 may play an important role in the parasite’s lytic cycle, which encompasses processes such as invasion, egress, and replication.

**Figure 5:**
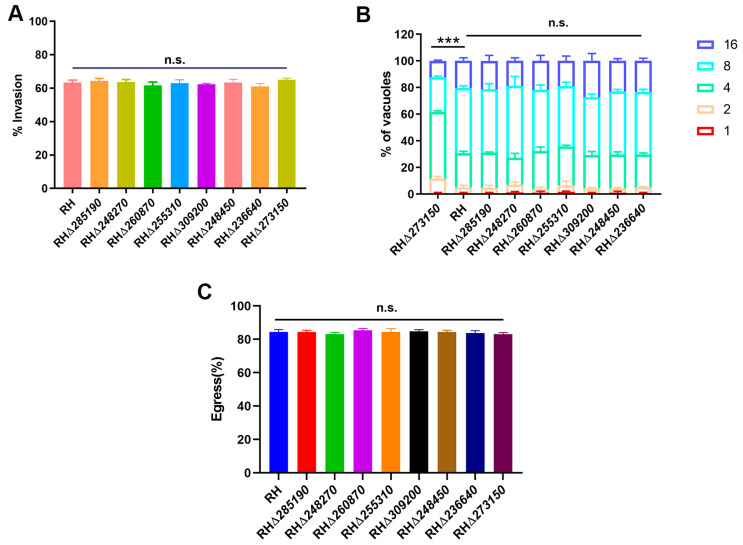
Invasion, intracellular replication, egress, and parasite virulence assays of RHΔ*zfp* strains and WT strains. (**A**) Compared with the WT strain, the invasion efficiency of eight RHΔ*zfp* strains showed no significant reduction. (**B**) HFF monolayers were infected with RHΔ*zfp* strains and the WT strain for 24 h. Parasitophorous vacuoles (PVs) containing 1, 2, 4, 8, and 16 tachyzoites were counted from 200 randomly selected PVs. Only the replication process of RHΔ*273150* showed significant inhibition compared to the WT strain. (**C**) No differences were detected in the egress efficiency between the eight RHΔ*zfp* strains and the WT strain. ***, *p* < 0.001, n.s., not significant.

To evaluate which stage of *T. gondii*’s lytic cycle was affected by disruption of TGME49_273150, we first investigated the impact of the RHΔ*zfp* strain on the early invasion process of *T. gondii* in host cells. The WT and RHΔ*zfp* strains were allowed to invade the HFF monolayers for 30 min, and invasion efficiency was determined. The results revealed that there were no significant differences in invasion efficiency in eight RHΔ*zfp* strains compared with the WT strain (*p* > 0.05), suggesting that the deletion of these eight *zfp* genes does not have a substantial impact on the invasion ability of *T. gondii* (Figure 5A).

Then, we evaluated the efficiency of intracellular replication and egress in both the WT and RHΔ*zfp* strains. At 23 h post-infection, the number of tachyzoites inside the PVs was counted. The results revealed that there were no significant differences in the intracellular replication between the seven RHΔ*zfp* strains and the WT strain (*p* > 0.05) (Figure 5B). However, the intracellular replication of the RHΔ*273150* was significantly reduced (*p* < 0.05) (Figure 5B). At 36 h post-infection, the HFF monolayers were treated with 3 mM calcium ionophore A23187 to induce egress, and the proportion of tachyzoite egressed from PVs was monitored, and the results showed that there were no significant differences observed between the eight RHΔ*zfp* strains and the WT strain in egress efficiency (Figure 5C). These findings indicated that TGME49_273150 may play a critical role in parasite replication.

### 3.5. The Eight zfp Genes Were Not Virulence Factors of the T. gondii Type I RH Strain

**Figure 6:**
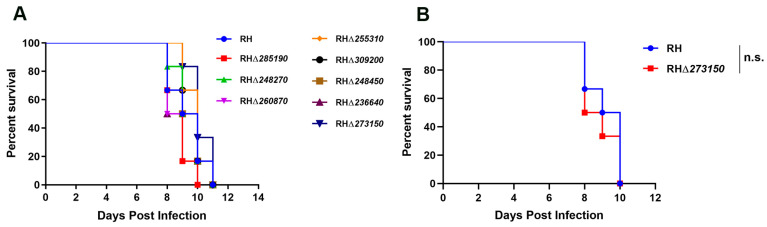
(**A**) Kunming mice (6 mice/strain) were injected intraperitoneally (i.p.) with 100 tachyzoites of the WT strain or the RHΔ*zfp* strains. (**B**) Compared with the WT strain, the survival of RHΔ*273150*-infected mice (50 tachyzoites) was not significantly different. The infected mice were monitored daily. n.s., not significant.

To assess whether the deletion of the eight *zfp* genes impacts the parasite’s acute virulence in vivo, female Kunming mice were intraperitoneally injected with 100 tachyzoites of both the WT strain and the RHΔ*zfp* strains, respectively. For TGME49_273150, due to its low phenotypic value, deletion resulted in slow growth of *T. gondii*. However, considering that the high virulence of the RH strain might mask the attenuating effect of the RHΔ*273150* strain, we conducted an additional virulence experiment on the RHΔ*273150* strain with half the number of tachyzoites (50 tachyzoites). The clinical symptoms of mice were monitored daily. Mice infected with the RHΔ*zfp* strains displayed a typical pattern of acute toxoplasmosis, consistent with those infected with the WT strain, and all infected mice reached a humane endpoint within 8–12 days of infection (Figure 6). Collectively, these results indicated that the eight *zfp* genes are not virulence factors for the type I RH strain of *T. gondii*.

## 4. Discussion

*T. gondii* infection poses a significant threat to human and animal health, and the development of safe and effective vaccines for both humans and animals remains a major challenge [22]. Previous studies have identified candidate genes for attenuated *T. gondii* vaccines using the CRISPR-Cas9 technology and evaluated their efficacy in protecting against *T. gondii* reinfection [23,24,25,26,27]. Nonetheless, these fall far short of what is needed for the development of a commercially viable anti-*T. gondii* vaccine with lasting protective efficacy. Elucidating the functions of *T. gondii* proteins will lay the foundation for researching *Toxoplasma* vaccines. ZFPs are extensively distributed in eukaryotic genomes and participate in diverse cellular functions [28]. *T. gondii* possesses at least 300 putative ZFPs; however, their specific roles remain insufficiently characterized [11]. This study explored the biological functions of eight *zfp* genes in *T. gondii*. By studying these genes and their associated proteins, we seek to gain insights into the pathogenic mechanisms of *T. gondii* and identify potential targets for effective vaccines and therapeutic interventions against toxoplasmosis.

ZFPs constitute an extensive eukaryotic family characterized by zinc finger motifs, encompassing the most common subfamilies such as C2H2-type, CCCH-type/C3H1-type, and C3HC4-type (RING finger) ZFPs [6]. The C2H2-type family comprises numerous transcription factors capable of binding to specific DNA regions, exerting vital regulatory functions in eukaryotes [7]. C3HC4-type (RING finger) ZFPs, which belong to another subclass of the ZFP superfamily, are present in various organisms and play important regulatory roles during different developmental stages [6,29,30]. The regulation of C2H2-type and C3HC4-type (RING finger) ZFPs under environmental stress has been extensively studied in plants [31,32,33,34]. However, studies of C2H2-type and C3HC4-type (RING finger) ZFPs in apicomplexan parasites are still limited. The best-studied C2H2-type ZFP of apicomplexan parasites is TgZNF2 [12]. The inducible knockout of TgZNF2 resulted in significant proliferation defects in the tachyzoites of *T. gondii*, while complementation with the homologous protein PfZNF2 effectively rescued the parasite’s proliferation defects, demonstrating that the pivotal function of TgZNF2 is highly conserved in *Plasmodium falciparum* [12].

CCCH-type/C3H1-type ZFPs constitute a class of RNA-binding proteins that play diverse regulatory roles throughout mRNA metabolism [6,35,36,37]. Nearly 60 types of CCCH-type ZFPs have been identified in both humans and mice, many of which function as regulators of immune responses and immune-related mechanisms (innate immunity activation) [38]. The functions of CCCH-type ZFPs have been characterized in various protozoa, particularly emphasizing their roles in the differentiation processes within trypanosomes [39].

In *Trypanosoma brucei*, for example, CCCH-type ZFPs such as TbZFP1, TbZFP2, and TbZFP3 are core components involved in normal differentiation stages of the parasite’s life cycle [40,41,42]. Knockout of the CCCH-type ZFP TcZC3H31 in *Trypanosoma cruzi* impairs the effective differentiation into an infectious subperiodic form [43]. Post-transcriptional regulation is a critical link in the cyclogeny of eukaryotes [44]. For instance, ZC3H11 in trypanosomes is an indispensable post-transcriptional regulator involved in various forms of trypanosome development and provides protection against heat shock [45]. TbZC3H20 exerts post-transcriptional regulation to stabilize transcripts linked to trypanosome development, namely mitochondrial carrier protein (MCP12) and trans-sialidase (TS-like E) [46]. *P*. *falciparum* expresses a CCCH-type ZFP known as ZNF4, which plays a role in regulating the sexual reproduction of the male gametophyte by controlling male gametophyte-related genes [47].

However, the functions of CCCH-type ZFPs in *T. gondii* have not been extensively characterized. The functions of only a few ZFPs were studied. Disruption of the TgZFP1 significantly reduced tachyzoite–bradyzoite differentiation, providing initial insights into essential factors involved in this process in *T. gondii* [10]. The recent identification of BFD1 as the master regulator of tachyzoite-to-bradyzoite differentiation in *T. gondii* has facilitated the characterization of another CCCH-type ZFP named BFD2. BFD2 acts as a downstream transcriptional activator of BFD1 and positively regulates the protein level of BFD1, providing insights into the differentiation process of *T. gondii* during chronic infection [14]. Given the crucial roles of CCCH-type ZFPs in different parasites, further exploration of this gene family will likely reveal their more extensive functions.

A lower CRISPR-based phenotypic value suggests a greater contribution to the parasite’s fitness, indicating its potential status as an essential gene [48]. In this study, seven *zfp* genes exhibit relatively high phenotypic values, which may suggest functional redundancy in cell culture. However, TGME49_273150 has a lower phenotypic value of −2.1, indicating its potential involvement in the lytic cycle of *T. gondii*. Signal peptides are short amino acid sequences that play a role in protein translocation and localization to the secretory pathway [49]. Among these examined ZFPs in the present study, none of the *zfp* genes were predicted to contain a signal peptide. Transcriptomic data analysis indicated that the expression of these eight *zfp* genes was not specific to a particular pattern and did not exhibit stage-dependent expression during the cell cycle. The differential expression of TGME49_255310 in different *T. gondii* strains suggests that the parasite might regulate this gene in a strain-specific manner.

In the present study, we initially examined the subcellular localization of the eight ZFPs in tachyzoites of *T. gondii* type I RH strain. The RH273150-HA protein was predominantly localized in the nucleus, while RH248270-HA, RH255310-HA, RH309200-HA, and RH236640-HA were observed in the cytoplasm. RH285190-HA, RH260870-HA, and RH248450-HA did not display specific localization fluorescence, possibly due to their low or undetectable expression at the tachyzoite stage. Additionally, Western blotting observed target bands corresponded to the expected size. However, RH255310-HA and RH309200-HA showed multiple bands in addition to the target band. The presence of these additional bands in Western blotting could be ascribed to post-translational modifications or protein degradation occurring during the experimental process.

Further investigation into the biological functions of these eight *zfp*s was conducted. Our results showed no significant differences in plaque size or a number between seven *zfp* mutant strains and the WT strain (*p* > 0.05). Our findings were in alignment with the high CRISPR phenotypic score, suggesting that these seven *zfp* genes are not essential for the growth of *T. gondii* in vitro [48]. It is worth noting that TGME49_260870 displayed the highest expression in day 0 (unsporulated) oocysts during sexual reproduction, suggesting that TGME49_260870 may plays a role in the sexual reproductive stage within the intestines of the definitive host rather than in the tachyzoite stage. Therefore, the roles of these genes in other life cycle stages of *T. gondii* require further investigation. Our study highlights TGME49_273150 as a significant gene in *T. gondii* that plays specific roles during its growth. Nevertheless, further investigation is required to elucidate the precise stage of the cell cycle at which TGME49_273150 inhibits *T. gondii*.

All infected mice died within 10–11 days, indicating that the eight *zfp* genes in the RH strain did not lead to significant changes in parasite virulence. However, *T. gondii* has a diverse spectrum of intermediate hosts, such as pigs, sheep, cattle, and other wild carnivores. Given the different expression patterns, this raises the possibility that these *zfp* genes might also influence virulence in these intermediate hosts, which warrants further investigations.

## 5. Conclusions

In summary, we successfully generated C-terminal epitope tagging strains and gene-deleted strains for eight *zfp* genes in the *T. gondii* type I RH strain. RH248270-HA, RH255310-HA, RH309200-HA, and RH236640-HA were found to localize in the cytoplasm, while RH273150-HA was expressed in the nucleus. RH285190-HA, RH260870-HA, and RH248450-HA showed undetectable in the tachyzoite stage. The deletion of seven *zfp* genes had no significant impact on *T. gondii* virulence in mice, while the disruption of TGME49_273150 significantly reduced the intracellular replication capacity of the RH strain. The deletion of a single *zfp* gene did not attenuate the virulence of the *T. gondii* type I RH strain in mice. Thus, it is necessary to explore the potential effects of double or triple *zfp* gene knockout combinations on the growth and virulence of the parasites, and further extensive mechanistic studies of *zfp* genes in *T. gondii* are warranted.

## Figures and Tables

**Table 1:** Bioinformatic features of zinc finger proteins (ZFPs) of *Toxoplasma gondii*.

Gene ID	Product Description	Exons	Phenotype Value	Mol wt (kDa)	TMHMMa *	Predicted Signal Peptide
TGME49_285190	zinc finger, C3HC4 type (RING finger) domain-containing protein	3	1.09	70.082	no	no
TGME49_248270	zinc finger (CCCH type) motif-containing protein	2	0.28	87.407	no	no
TGME49_260870	zinc finger cdgsh type protein	4	0.92	21.367	no	no
TGME49_255310	zinc finger (CCCH type) motif-containing protein	1	0.7	91.510	no	no
TGME49_309200	zinc finger (CCCH type) motif-containing protein	1	0.41	101.661	no	no
TGME49_248450	zinc finger, C3HC4 type (RING finger) domain-containing protein	8	0.28	86.828	yes	no
TGME49_236640	zinc finger, C3HC4 type (RING finger) domain-containing protein	7	0.61	55.618	yes	no
TGME49_273150	zinc finger, C3HC4 type (RING finger) domain-containing protein	2	−2.1	78.603	no	no

* Prediction of transmembrane helices was performed using the TMHMM program version 2.0.

## Data Availability

The original contributions presented in this study are included in the article. Further inquiries can be directed to the corresponding authors.

## Supplementary Materials

The following supporting information can be downloaded at https://www.mdpi.com/article/10.3390/pathogens12101232/s1. Table S1: Primers used in the construction of the epitope-tagging strains; Table S2: Primers used in the construction of the *zfp* genes knock-out strains.
