# Supplementary material for: Functional Characterization of Eight Zinc Finger Motif-Containing Proteins in Toxoplasma gondii Type I RH Strain Using the CRISPR-Cas9 System
Source: Pathogens. 2023 Oct 11;12(10):1232. doi: 10.3390/pathogens12101232 (PMC10609756; doi:10.3390/pathogens12101232)
Supplement: Supplementary file 1 [file pathogens-12-01232-s001.zip › pathogens-2616390-supplementary.pdf]

Table S1: Primers used in the construction of the epitope-tagging strains.

Gene	sgRNA	HR-primers (5′-3′)	KO-primers (5′-3′)
TGME49_285190	GGCTGTCGACAGCAGTGAAG	Forward:CGGCGCGGCTTCGACGGTCGGGAAGACGGCTGTCGACAGCAG	Forward:CTTCATCGCCGCCCTTCTTCG
		Reverse:AGGACCGCCACAGGGACCGGCGGAGAAAACTTTTCACCGCTT	Reverse:CCACAACAACAGGACCGCCACA
TGME49_248270	GCTCGCGGCTAGGAGCCCAT	Forward:GGCGCCGCAGGGGGCGCTGGGGCTGAGACGCCCGCTCGCGGC	Forward:CCTGGGGCACGAACGAGGAG
		Reverse:CGGCTCCCCTCTCGTGGACAACTGGGACTTTGCCCTCCAATG	Reverse:GGAAACGAGTCGGACGGTTATTGA
TGME49_260870	ACCGTCCACCCCCGCAGTCG	Forward:TCTGACGTTGCGACCGCCACCCTCTCTGCTCGCTACAGCCTC	Forward:TGGATTTTCCTGCCTTTCGCTCTG
		Reverse:TGGAGGGAGGAAGCATTTTGCCGCTTGTTCGTTGTTCCTCGA	Reverse:GCATTTTGCCGCTTGTTCGTTG
TGME49_255310	GCAACAGCCTCGACGTGATG	Forward:GAAGCAAGGAAACCTCCGCAAGAGCGGGCAACAGCCTCGACG	Forward:GCAACCGAAGCTACACGAGCAAT
		Reverse:CTTGCACCTCCACATGTCTTCCTTCGTCGAGATAATCCACAT	Reverse:GCACAAGCCACCTCACATACCACA
TGME49_309200	CTTTGTTTTACGAAGATTAG	Forward:ACGGAGGAGCTCAATGCAGCAGCGCCTTTGTTTTACGAAGAT	Forward:ATCCGCTTCCACATTCGACTCAG
		Reverse:TCAATCCCGTCGTACCATTCTCTGCCATCTTCCACTCCCCTA	Reverse:TCCCCACGCACCTCTTCACAG
TGME49_248450	CCTGCCTCCATTCCCTTGAG	Forward:GAGTGCTCTGTGTACACTTCAGGTGCCCTGCCTCCATTCCCT	Forward:CGGCCTTCGGCGAGACTTCC
		Reverse:CTACCTTTCCGAATATCGGAGAGGCCGGTTGTTTGTCCGCTC	Reverse:GGTGCTTATTGTGCGACAGATTGG
TGME49_236640	AAGAGAACGGGAGCACTTGA	Forward:CTGCGTATTCCAGGCACGGCGGCGCAAGAGAACGGGAGCACT	Forward:TACGAAGCATCGACGAGCCGTTTG
		Reverse:TCCGCACCTGCCCTCAGCCTTTTTCCCATACGCCTTCCATCA	Reverse:TCCGCACCTGCCCTCAGCCT
TGME49_273150	AGTTGCTGAAGCAAACGTGA	Forward:GGCAAGCGCATGCACCACGCGGTCGAGTTGCTGAAGCAAACG	Forward:GGAGTCTCCGCTGACTCTGTCGC
		Reverse:TCCCCGCCTGCTCCTCTGTCCGCATGTCGAGACGCTCCATCA	Reverse:TCATTTCGTCTTATCCGCCGTTTT

Table S2：Primers used in the construction of the *zfp* genes knock-out strains.

Gene	sgRNA	KO-primers (5′ -3′)	U5-Gibson-primers (5′-3′)	U3-Gibson-primers (5′ -3′)
TGME49_285190	GTGCGAAGCTGCAAGTGAAG	Forward：ACTGACGACGCTGCGTACAACCA	Forward：GACCTCATACCGCCTCCACTTCTC	Forward：CGATCCAGAAACAACGCAGCAA
		Reverse：GCCACTAGCCACCCACGAATAGG	Reverse：TGCTGTTGTTGTTCCCCATTTCC	Reverse：TGTCCAGAGTCAGGGACATCCACA
TGME49_248270	GACTGGCGAAGAGAAGACGA	Forward：GTGCAGGAAACCAGAAGGGTCG	Forward：TGGTTCCTTTATTCGCCCTTGGT	Forward：GTTCCCCTTCTCGCCTGGTCG
		Reverse：GCAACAGGGGTGTTCGGGGAG	Reverse：GCAGATACACTTGGCTTTCCTCCC	Reverse：CCGTTCCCTTCACTAGCCCTTTCA
TGME49_260870	GCGAAGCTCCGCGACAGTCG	Forward：CCTCCCGCCCGTCAGGTCAT	Forward：CCCAGGTGGACGGAGATGTTGC	Forward：GAAATGGATTTTCCTGCCTTTCGC
		Reverse：CGCAGAGCGGCAACATTCTTCAA	Reverse：TGCGGAAGTTTCTTGGTGTTGATG	Reverse：CGCCACCGTCGGATCTCAAC
TGME49_255310	GGTCGACATAGACTCGGAGG	Forward：CCGCATTCCCCTTCCTCACC	Forward：TGAGGCTCATCGCTGGATATTTTG	Forward：GACGAAGCGGAAGCAGGACATC
		Reverse：GCGACGAAGGCGTTCTGGTG	Reverse：GGGAGGCTGTGGGCTCGTCT	Reverse：AAACCACCTTCTCCAGGAACGACA
TGME49_309200	GGCGCGGTCCATCCTGGTGG	Forward：CGACTTCCACTGGGTCTTCGTACC	Forward：AGGCATGTTCACTCGCACAGGTT	Forward：GGCCTCGATGGGGTCTTATTGG
		Reverse：CCAGGCAGCGAACCATCAGC	Reverse：CGCTCGTAGGCGATGGAAGG	Reverse：CCGAAGGAAAAGTCAGCGTGGTG
TGME49_248450	GTGTCGTCCTGTACTTCGTG	Forward：TTCTGTCGATTTCTTTGCTGTGCG	Forward：GAAGGTGCCCTTTTCACCAAACC	Forward：TTCTTGCTTGGATGGTCAGATGGC
		Reverse：CATGGCGTGATGGACCTGTCTG	Reverse：CGGCGTACCGGAGCTATCCA	Reverse：CCTTGAAAATTGTTGGCAGGGTG
TGME49_236640	GCTGCCGCTCCTGACATACG	Forward：GCTACGGCTGGTTTATTCTCGGTG	Forward：AGGAAAAGCTGAGGATTTTCGTGC	Forward：CTTGCCCATTTTGTGGTGTTTCG
		Reverse：CGTCTTCCTTCTTGCCTTTGCTTC	Reverse：CTCGCCGTAGAGTGTCTGTTGTGG	Reverse：CGCAGTCAATTTGCTTTTGGTCTC
TGME49_273150	GCTGGTGCCTGACCAGGCTC	Forward：GCTTGGTCGAGTGTCTCCTCCG	Forward：AGAAACTCCGCCCACGGTTCTG	Forward：CGGCTCGTCTGCTTCCTGTCTT
		Reverse：CCGATCCTTTCGTTCTGCGTCTT	Reverse：TCCCTCCCTGCTGCTCCTCC	Reverse：GAGCTTTCCTGCGACGGGACT
